# Bioinspired Membranes with Silver Sulfadiazine and Piperine for Enhanced Cutaneous Permeability

**DOI:** 10.1021/acsomega.5c08994

**Published:** 2025-10-29

**Authors:** Gabriely Cristini Batista de Deus, Heloisa Diehl Doring, Yara Schuvinski Ricken, Marta Elisa Rosso Dotto, Diego Galvan, Tatiana Herrerias, Eloah Latocheski, Camila Fabiano de Freitas

**Affiliations:** † Department of Chemistry, 28117 Federal University of Santa Catarina, Florianópolis, Santa Catarina 88040-900, Brazil; ‡ Department of Physics, Federal University of Santa Catarina, Florianópolis, Santa Catarina 88040-900, Brazil; § Department of Clinical Analysis, Federal University of Santa Catarina, Florianópolis, Santa Catarina 88040-900, Brazil

## Abstract

Developing bioinspired membranes for effective wound healing, particularly for burn injuries, significantly advances biomedical materials. In the present study, we focused on the design and optimization of membranes based on chitosan (CT) and Pluronic F127, incorporating silver sulfadiazine (SSD) for antimicrobial activity and piperine (PIP) as a permeation enhancer. Using a design of experiments with desirability, the optimal membrane composition was determined to be 20 mg/mL CT, 10 mg mL^–1^ F127, 0.050 mg mL^–1^ SSD, and 0.050 mg mL^–1^ PIP, which exhibited excellent homogeneity, mechanical stability, and controlled drug release properties. The membranes were characterized using FTIR, DSC, TGA, AFM, and contact angle measurements, revealing high thermal stability, moderate hydrophilicity, and pH-responsive dissolution behavior. The membranes demonstrated significant water absorption and swelling degree (SD > 500%), creating a moist environment conducive to tissue regeneration. Ex vivo porcine skin permeation studies showed that incorporating PIP more than doubled SSD deposition in both the epidermis (9.82 → 21.75 μg g^–1^) and dermis (2.24 → 4.99 μg g^–1^). In vitro microbiological assays against *Escherichia coli* and *Staphylococcus aureus* (ATCC and clinical strains) revealed that SSD-containing membranes significantly reduced bacterial viability, with the complete membrane (SSD + PIP) showing the strongest inhibition. For *E. coli* clinical strains, the addition of PIP enhanced the bactericidal effect of SSD (76% vs 33% reduction), consistent with its reported role as an efflux pump inhibitor. Overall, these multifunctional membranes combine sustained SSD release, improved and reproducible skin permeation, and enhanced antimicrobial efficacy in the evaluated strains, offering a promising platform for advanced wound dressings in burn care and other skin injuries.

## Graphical abstract

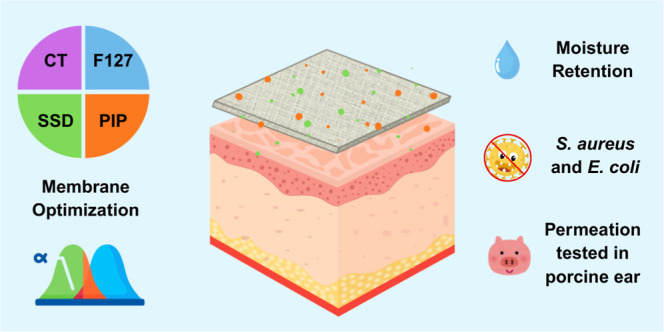

## 1 Introduction

Skin wounds, particularly those caused by burns, represent a significant global health challenge, with approximately 180,000 cases reported annually, according to the World Health Organization (WHO). Burn injuries are especially prevalent in low- and middle-income countries, where access to advanced medical care is often limited.^1^ These injuries not only cause severe physical trauma but also lead to complications such as infections, prolonged healing times, and significant psychological distress. Effective wound management is therefore critical to reducing morbidity and improving patient outcomes. Traditional wound dressings, such as gauze and passive bandages, provide basic protection but cannot actively promote healing or prevent infections. This limitation has driven the development of bioactive wound dressings, which interact with the wound bed to create an optimal environment for tissue regeneration.^2–4^

Among the various materials explored for advanced wound care, chitosan (CT), a natural biopolymer derived from chitin, has gained considerable attention due to its unique properties.^5,6^ CT is biocompatible, biodegradable, and exhibits inherent antimicrobial activity, making it an ideal candidate for wound healing applications. Its ability to form films and gels further enhances its utility in creating flexible and functional wound dressings.^7^ However, CT-based materials often have limitations such as low mechanical strength and rapid dissolution in acidic environments.^8^ To address these challenges, CT is frequently combined with synthetic polymers, such as Pluronic F127, a triblock copolymer composed of poly­(ethylene oxide) and poly­(propylene oxide). Pluronic F127 improves the mechanical properties of CT-based membranes and enables responsive drug release, making it highly suitable for biomedical applications.^9^

Incorporating silver sulfadiazine (SSD), a widely used antimicrobial agent, further enhances the therapeutic potential of these membranes. SSD is considered the gold standard for burn treatment due to its broad-spectrum antimicrobial activity, which effectively inhibits the growth of bacteria such as *Pseudomonas aeruginosa* and *Staphylococcus aureus*.^10^ However, the use of SSD is often limited by its poor solubility and potential cytotoxicity at high concentrations.^11^ To overcome these limitations, integrating SSD into CT matrices and Pluronic formulations allows for controlled release, reducing the risk of toxicity while maintaining antimicrobial efficacy. For instance, a formulation composed of SSD, CT and sodium alginate reduced wound width by 75% compared to the commercially available cream.^12^ Moreover, SSD-loaded CT nanoparticles were able to enhance antimicrobial activity by inhibiting growth of Gram positive, Gram negative, and Candida in burn wounds.^13^ When employed in films for wound dressing, Fajardo et al. demonstrated the CT material was capable of sustain the release of SSD for up to 96 h at physiological pH.^14^ Systems combining CT and Pluronic F127 can potentially further enhance this control considering F127 significantly enhanced drug delivery properties of SSD, improved mechanical properties and bactericidal action in previously developed hydrogels.^15,16^ Additionally, the inclusion of piperine (PIP), a bioactive alkaloid derived from black pepper, has been shown to enhance drug permeation through the skin. Piperine acts as a permeation enhancer by disrupting the lipid structure of the stratum corneum, thereby improving the bioavailability of incorporated drugs.^17^ In fact, curcumin, a compound with inherently poor bioavailability caused by limited aqueous solubility, had its permeation rate increased in about 1.9-fold from membranes designed for transdermal delivery due to the incorporation of PIP in the matrix.^18^

In light of the aforementioned, this study focuses on developing and optimizing bioinspired membranes composed of CT and Pluronic F127, incorporating SSD and PIP for the treatment of burn wounds. The membranes were prepared using a solvent casting method,^19^ and their composition was systematically optimized through a design of experiments. This approach enabled the identification of the optimal combination of CT, Pluronic F127, SSD, and PIP, ensuring homogeneity, mechanical stability, and optimized drug deposition properties.

## 2 Materials and Methods

### 2.1 Materials

Medium molar mass chitosan (CT, CAS 9012-76-4), with a degree of deacetylation of 87% and a viscosimetric molar mass of 10.6 × 10^4^ g mol^–1^, was used. Silver sulfadiazine (SSD, 98% purity, CAS 22199-08-2), piperine (PIP, 92% purity, CAS 94-62-2), Pluronic (F127, 99% purity, CAS 9003-11-6), Brain Heart Infusion (BHI), and 2,3,5-triphenyltetrazolium chloride (TTC) were purchased from Sigma-Aldrich. Acetone, acetonitrile, acetic acid, potassium chloride, sodium chloride, ethanol, propylene glycol, dibasic sodium phosphate, and monobasic potassium phosphate were purchased from NEON. Hydrogen Peroxide 35% was purchased from Exôdo Científica. Nitric Acid P.A. was purchased from LAFAN-Química Fina. Ultrapure water was used in all experiments. All reagents and solvents were of analytical grade and used without prior purification.

### 2.2 Preparation of Chitosan/Pluronic F127 Membranes

The polymeric membranes of CT and Pluronic F127, incorporated with SSD and PIP, were obtained by the solvent casting method.^19^ The F127 solutions were prepared in a heating bath (∼50 °C) under magnetic stirring at 300 rpm for 1 h, while the CT solutions were prepared at room temperature in an aqueous medium containing 1% (V/V) acetic acid, under mechanical stirring at 700 rpm for 24 h.^20^ SSD and PIP were dissolved separately in 2.5 mL of F127 solution, under magnetic stirring at 500 rpm for 30 min. The CT, F127, SSD, and PIP variables were evaluated using a full factorial design 2^4^ with triplicates at the center point, as presented in Table S1.

The CT solutions were transferred dropwise into the Pluronic F127 solutions (containing 2.5 mL of PIP and 2.5 mL of SSD). The solution containing all active ingredients was stirred for 1 h at 700 rpm to ensure uniformity of the liquid medium. Then, the film-forming solutions were transferred to 5 cm polystyrene Petri dishes and left to rest in an air-circulating oven at 40 °C for 24 h, ensuring adequate membrane formation for subsequent application.

The response was assessed based on (i) membrane thickness, (ii) weight, (ii) uniformity/presence of defects and/or cracks, and (iv) the sample’s crystallinity level. To this end, all samples were first subjected to visual and photographic analysis using the rear camera of a Samsung Galaxy S21 smartphone, with a resolution of 12 MP (wide angle), 12 MP (ultrawide angle), and 8 MP (telephoto). The samples were then evaluated by polarized light microscopy (PLOM). Then, the simultaneous analysis of the responses was performed using the desirability function proposed by Derringer & Suich.^21^ This tool aims to identify a common region that simultaneously satisfies all responses within the experimental domain. The models fitted for each response were evaluated regarding the quality of fit to the data. Subsequently, the estimated response values for each variable were transformed into individual desirabilities, which were then combined using the geometric mean, yielding the global desirability (D). For more details, see Galvan & Bona.^22^

The membrane formulation that exhibited the most favorable characteristics (CT/F127/PIP/SSD), namely uniform thickness, absence of structural defects, and lack of crystalline domains, was selected for further investigation through complementary characterization techniques. For comparative purposes, control membranes were also prepared under the same conditions. These included CT/F127, CT/F127/PIP, and CT/F127/SSD formulations, in addition to single-component membranes composed exclusively of CT or F127.

### 2.3 Thickness

The thickness of all membranes was measured with a digital micrometer (Marathon), calculating the arithmetic mean of three points: one central and two on the sides of each membrane.

### 2.4 Polarized Light Optical Microscopy (PLOM)

Polarized light optical microscopy (PLOM) was able to provide detailed information about the morphology and dispersion of compounds in the membranes, highlighting the presence or absence of crystalline particles, which appeared as luminous spots in the images.

Birefringence is the phenomenon responsible for these luminous spots when crystals are present. As crystals have different refractive indices, allowing light to split into two cross-polarized rays. Crystallinity was determined by visually observing the birefringence of the crystals (when present) in contrast to the amorphous regions, which showed no birefringence.

Membrane samples were cut into small sections and mounted on glass slides. Analyses were performed using an Olympus BX53 optical microscope equipped with polarizers and a halogen lamp. A digital camera (Olympus DP73) coupled to the microscope was used for image acquisition, and images were processed with CellSens software (Olympus). For each membrane, at least five different regions were examined to ensure representative analysis.

### 2.5 Atomic Force Microscopy (AFM)

Micrographs were obtained using a Nanosurf FlexAFM easyScan 2 Controller atomic force microscope (AFM)-Switzerland. The equipment was operated in intermittent contact mode, using a TAP190Al-G cantilever (radius <10 nm), with a resonance frequency of 190 kHz and a spring constant of 48 N m^–1^. Images were captured at a scanning frequency of 1 Hz and a resolution of 512 × 512 pixels, and were subsequently processed using WS × M 5.0 software, which is freely available online.^23^ In addition to evaluating the morphological characteristics of the sample surface, properties such as the roughness essential for the desired application were also investigated.

### 2.6 Fourier Transform Infrared Spectroscopy (FTIR)

Fourier transform infrared spectroscopy (FTIR) was used to evaluate the interactions between membrane components. Individual samples of CT and Pluronic F127, as well as CT/F127, CT/F127/PIP, CT/F127/SSD, and CT/F127/PIP/SSD membranes, were analyzed. Attenuated Total Reflection (ATR) was used to characterize the membranes, while for Pluronic F127 only, analysis was performed using a KBr pellet. Measurements were performed in absorbance mode, in the wavenumber range of 4000–400 cm^–1^, with a resolution of 2 cm^–1^ and 60 scans to ensure high data sensitivity.

### 2.7 Thermogravimetric Analysis (TGA)

Thermogravimetric analyses (TGA) were performed using a Shimadzu TGA-50 thermogravimetric analyzer, operating in a controlled nitrogen atmosphere with a 50 mL min^–1^ flow rate. Six mg of each sample was heated at a constant rate of 10 °C min^–1^, reaching a final temperature of 700 °C. The samples were stored in platinum cells during the experiments, providing thermal stability and minimizing potential interference during mass loss measurements. The mass loss (%) was recorded as a function of temperature, and derivative thermogravimetric (DTG) curves were generated to determine the onset temperature of degradation and the corresponding thermal events.

### 2.8 Differential Scanning Calorimetry

Thermal behavior of the samples was analyzed by differential scanning calorimetry (DSC), performed on a TA Instruments Q20. Approximately 2 mg of each sample of each sample was analyzed over a temperature range of 0–200 °C, with a heating rate of 10 °C min^–1^ in a nitrogen atmosphere.

### 2.9 Wettability Analysis by Contact Angle Measurements

The wettability and hydrophilicity of the membrane surfaces were evaluated by static water contact angle measurements using a goniometer equipped with a high-resolution camera (ramé-Hart 250). Membrane samples were affixed to glass slides using double-sided adhesive tape and positioned vertically in the instrument’s sample holder. Water droplets were carefully deposited onto the membrane surface using a disposable Pasteur pipet, ensuring consistent spacing between droplets. Upon deposition, images were recorded at a rate of 10 frames per second, and contact angle values were automatically calculated by instrument software using the sessile drop method. All measurements were performed at room temperature.

### 2.10 Determination of Membrane Dissolution Degree

The dissolution degree of the membranes was evaluated in solutions of acetate buffer (pH 5.5) and phosphate buffer (pH 7.4), simulating different physiological conditions. Membrane discs with a diameter of 1.5 cm were prepared using a circular punch, weighed on a high-precision analytical balance (±0.0001 g), and individually transferred into Falcon tubes containing 25 mL of the respective buffer solution. The samples were incubated in a thermostatic water bath at 37 ± 0.5 °C for 24 h.

After incubation, the discs were carefully removed, gently blotted with absorbent paper to remove surface moisture, and placed in a desiccator for 48 h to eliminate residual water. The dried samples were then weighed again. The dissolution degree (%DD) was calculated using eq 1

1 $$\%DD=\frac{m_{i}-m_{f}}{m_{i}}\times 100$$

where *m*_*i*_ is the initial dry mass of the membrane and *m*_f_ is the final mass after 24 h.

### 2.11 Determination of Membrane Swelling Degree

The swelling behavior of the membranes was evaluated to determine the water absorption capacity of the polymer matrices, a key property for wound dressing applications due to the presence of exudates and fluid exchange in injured skin.

The swelling degree was determined gravimetrically. Membrane samples were cut into discs with a diameter of 1.5 cm and immersed in 25 mL of phosphate buffer solution (pH 7.4) in individual beakers. The experiment was conducted at 37 ± 0.5 °C under constant stirring in a thermostatic water bath.

Samples were removed from the buffer at predetermined time intervals, gently blotted with absorbent paper to remove surface moisture, and immediately weighed using an analytical balance. After weighing, the samples were returned to the buffer solution. Measurements were taken every 5 min during the first hour, followed by hourly measurements up to 3 h. Additional weighings were performed after 23, 24, and 72 h to assess mass consistency and equilibrium swelling. The swelling degree (%SD) was calculated using eq 2

2 $$\%SD=\frac{m_{t}-m_{0}}{m_{0}}\times 100$$

where *m*_0_ is the initial dry mass of the membrane, and *m*_*t*_ is the mass at time *t*. All experiments were performed in triplicate to ensure reproducibility.

### 2.12 Mechanical Analysis

The mechanical properties of the polymeric membranes, including maximum tension and modulus of elasticity, were investigated through tensile versus deformation tests. A universal mechanical Texture Analyzer model TA.HD Plus (Stable Micro Systems, UK) was equipped with a load cell of 50 kgf capacity. Membrane samples were cut into 6.0 cm × 1.5 cm strips.

Each sample was mounted vertically between the upper and lower grips, ensuring a uniform gauge length. The test was conducted at a crosshead speed of 0.8 mm/s until rupture. The analysis was conducted at room temperature and following the guidelines of ASTM D882 (2002).^24^

The instrument recorded stress (kg/mm^2^) vs strain (%) data in real time. Triplicate measurements (*n* = 3) were performed for each formulation, and the results were expressed as mean ± standard deviation.

### 2.13 Ex Vivo Porcine Skin Drug Deposition Studies

Drug deposition studies were performed using porcine ear skin model. The skin preparation followed the method described in the literature.^26,27^ The ears did not undergo thermal or scraping processes to avoid possible abrasions. Membranes were cut into discs with a diameter of 2.5 cm and were placed above epidermis. Then, 500 μL of PBS was added on top of the membrane to facilitate permeability. After 24 h, the membranes were removed, the skin was repeatedly washed with purified water, and the dermis and epidermis were subsequently separated. Skin layers were then digested using nitric acid and hydrogen peroxide, and the silver content in both layers was quantified using Flame Atomic Absorption Spectrometry (PerkinElmer- pinAAcle 900T).

### 2.14 Microbiological Assay

Microbiological assays were carried out using ATCC reference strains, namely *S. aureus* (ATCC 25823) and *Escherichia coli* (ATCC 25922). In addition, multidrug-resistant clinical strains of *S. aureus* and *E. coli* were used, which were kindly provided by the Applied Molecular Microbiology Laboratory (MIMA) of the Federal University of Santa Catarina (UFSC).

The antibacterial activity was evaluated by the disk diffusion method and microplate trials. The sensitivity profile of the multidrug-resistant clinical bacterial strains was determined according to the protocol established by the Brazilian Committee on Antimicrobial Susceptibility Testing (BrCAST).^25^ The inhibition halos formed around the samples were measured to assess the antimicrobial effect. The bacteria were maintained in brain heart infusion (BHI) broth for reactivation and growth at 37 °C for 24 h and subsequently cultured on Muelle Hinton Agar for colony development. From these colonies, a bacterial suspension was prepared in sterile saline solution to achieve a turbidity standard of 0.5 on the McFarland scale, corresponding to a concentration of 1.5 × 10^8^ CFU/mL. This suspension was diluted 1:10^4^ in BHI, and 2 mL of the dilution was added to the wells of a microplate (12 wells), followed by incubation at 37 °C for 24 h to allow biofilm formation.

The treatment protocol was conducted using the complete membrane (CT/F127/PIP/SSD), along with the respective control groups: SSD, CT/F127, CT/F127/PIP, and CT/F127/SSD. As a negative control, the blank chitosan-Pluronic membrane without SSD or PIP was employed (CT/F127). For positive control, discs containing 25 μm of Trimethoprim + Sulfamethoxazole (SUL) were applied twice, with a 24 h interval between applications. To assess bacterial cell viability, the reagent TTC (2,3,5-triphenyltetrazolium chloride) was added, and the plates were incubated for 2 h. Following this period, readings were performed using a microplate spectrophotometer (BioTek, Epoch microplate spectrophotometer) at 540 nm. Three replicates were conducted for each treatment group. The absorbance results were converted to bacterial cell viability percentages using eq 3

3 $$bacterial\;cell\;viability\;\left(\%\right)=\frac{Absorbance\;Sample}{Blank\;Absorbance}\times 100$$

Statistical analyses were performed using GraphPad Prism (version 8.4.0). Data were expressed as mean ± standard error of the mean (SEM). Comparisons were carried out using paired *t*-test and one-way ANOVA followed by Games-Howell post hoc test, when appropriate. A significance level of *p* < 0.05 was considered statistically significant.

## 3 Results and Discussion

### 3.1 Preliminary Morphological Characterization for Optimization

All 19 samples obtained from the 2^4^ (Table S1) full factorial design were initially evaluated. Quantitative analyses were performed through measurements of membrane thickness and weight, while qualitative assessments relied on photographic inspection for uniformity, defects, and/or cracks. In addition, the degree of crystallinity of the samples was determined. The corresponding results are presented in Table 1. The DOE optimization focuses on composition (CT, F127, SSD, PIP concentrations). However, process parameters such as drying temperature, solvent ratios, and casting conditions can also influence membrane uniformity, crystallinity, and drug release. Although these parameters were not systematically varied in the present study, they were carefully selected based on preliminary screening experiments, ensuring uniform and reproducible membranes with optimal formation and functionality.

**Table 1:** Two-Level Factorial Design with Three Replicates of the Central Point^a^

	independent variables				dependent variables				
runs	[CT]/mg mL^–1^	[F127]/mg mL^–1^	[SSD]/mg mL^–1^	[PIP]/mg mL^–1^	uniformity level	crystallinity level	presence of defects and/or cracks	thickness/mm	weight/g
1	–1 (10)	–1 (10)	–1 (0.05)	–1 (0.05)	0	1	1	0.0307	0.1139
2	+1 (20)	–1 (10)	–1 (0.05)	–1 (0.05)	1	2	1	0.0417	0.1697
3	–1 (10)	+1 (20)	–1 (0.05)	–1 (0.05)	0	2	0	0.0403	0.0942
4	+1 (20)	+1 (20)	–1 (0.05)	–1 (0.05)	0	2	1	0.0473	0.2120
5	–1 (10)	–1 (10)	+1 (0.2)	–1 (0.05)	0	0	1	0.0230	0.1074
6	+1 (20)	–1 (10)	+1 (0.2)	–1 (0.05)	1	1	1	0.0343	0.1738
7	–1 (10)	+1 (20)	+1 (0.2)	–1 (0.05)	0	2	0	0.0000	0.0000
8	+1 (20)	+1 (20)	+1 (0.2)	–1 (0.05)	0	2	1	0.0413	0.1758
9	–1 (10)	–1 (10)	–1 (0.05)	+1 (0.15)	0	1	1	0.0267	0.1136
10	+1 (20)	–1 (10)	–1 (0.05)	+1 (0.15)	1	1	1	0.0340	0.179
11	–1 (10)	+1 (20)	–1 (0.05)	+1 (0.15)	0	0	0	0.0000	0.2252
12	+1 (20)	+1 (20)	–1 (0.05)	+1 (0.15)	0	1	1	0.0500	0.2177
13	–1 (10)	–1 (10)	+1 (0.2)	+1 (0.15)	0	1	1	0.0116	0.1187
14	+1 (20)	–1 (10)	+1 (0.2)	+1 (0.15)	1	0	1	0.0210	0.1886
15	–1 (10)	+1 (20)	+1 (0.2)	+1 (0.15)	0	2	0	0.0457	0.1056
16	+1 (20)	+1 (20)	+1 (0.2)	+1 (0.15)	0	1	1	0.0457	0.2281
17	0 (15)	0 (15)	0 (0.125)	0 (0.1)	0	1	0	0.0307	0.1576
18	0 (15)	0 (15)	0 (0.125)	0 (0.1)	0	1	1	0.0353	0.1646
19	0 (15)	0 (15)	0 (0.125)	0 (0.1)	0	1	1	0.0397	0.1625

a For uniformity, 1 denotes homogeneous membranes and 2 denotes heterogeneous membranes. For crystallinity, 0 denotes crystals <10 μm, 1 denotes crystals >10 and <100 μm, and 2 denotes crystals >100 μm. For the presence of defects and/or cracks, 0 denotes absence and 1 denotes presence.

#### 3.1.1 Thickness and Weight

The quantitative analyses were performed considering the weight and thickness of the membranes. The thickness was measured using a micrometer at three points on each membrane: the center point and the lateral ends. Each membrane’s arithmetic mean was calculated (see Table S2 in the Supporting Information).

Analysis of the thickness data reveals those membranes with the lowest standard deviation (e.g., No. 6, with a deviation of 0.001 mm—Table S2) exhibit greater homogeneity, indicating a uniform distribution of components across the entire surface. This uniformity correlates with the structural stability and cohesion of the polymer matrix, which is also reflected in a lower propensity for fracture. In contrast, membranes with high deviations (such as No. 13, with a deviation of 0.012 mm—Table S2) present a less uniform structure, possibly due to phase separation between CT and Pluronic F127. This segregation contributes to the formation of weak regions that compromise mechanical strength, making these membranes more susceptible to fracture.

Furthermore, mass variations between membranes indicate possible differences in residual solvent content or moisture absorption, especially in membranes with higher masses than expected for the solid components. Membranes such as No. 4 and No. 16, which have masses of 0.212 and 0.228 g, respectively, may have retained a greater amount of solvent, resulting in variations that influence both membrane thickness and density. This retention can negatively affect homogeneity, especially in formulations where complete solvent evaporation is crucial to achieving a cohesive and uniform structure. Given the above, based on the analysis of the thicknesses of the membranes produced, the average values obtained ranged from approximately 0.021 mm to 0.056 mm. In comparison, the thickness of the human epidermis generally ranges from 0.05 mm to 1.5 mm, depending on the area of the body and hydration status.^28^ The thinnest membranes, such as membrane No. 5 (0.023 mm), approximate the thickness of the most superficial layer of the epidermis, which varies between 10 and 20 μm in thin areas. This similarity in thickness suggests that bioinspired membranes may be viable for cutaneous application, promoting more efficient adaptation to the skin’s topography, which may facilitate drug permeation and localized release. Furthermore, the thickest membranes, close to 0.05 mm, approximate the lower limit of the epidermis, making them promising for mimicking epidermal barriers and enhancing protection and sustained drug release.^29^

#### 3.1.2 Homogeneity and Fragility Analysis

This analysis observed aspects such as homogeneity, heterogeneity, weight, and tendency to break/fragility, as illustrated in Figure 1.

**Figure 1:**
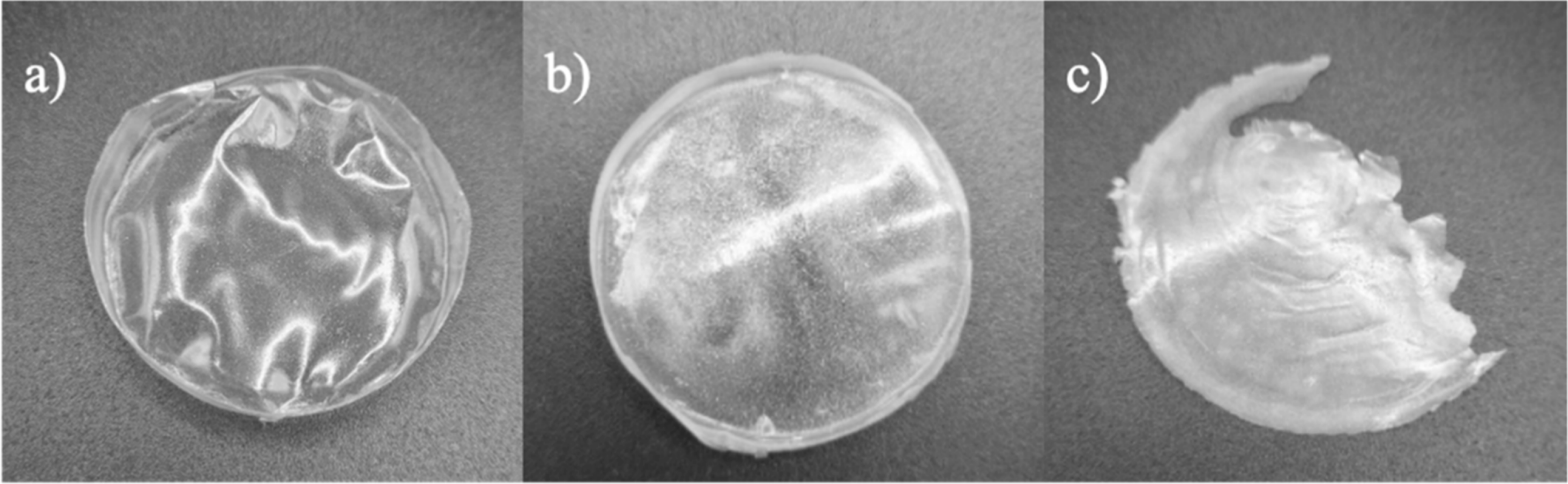
Photographs of the groups of membranes obtained (a) homogeneous membrane, (b) heterogeneous membrane, and (c) heterogeneous and brittle membrane.

The homogeneous membranes are represented by level 1 in Table 1 (No. 2, 6, 10, 14—Table S2 and Figure 1a). These membranes presented uniform component distribution, mainly due to the balanced interaction between CT and Pluronic F127. CT, a cationic biopolymer, shows good affinity for acidic aqueous solutions due to the protonation of its amino groups (p*K*_a_ 6.3)^5^ and its ability to form hydrogen bonds, providing a cohesive matrix that facilitates the homogeneous dispersion of F127.^30^ In turn, Pluronic F127, a triblock copolymer with amphiphilic properties, tends to organize into stable micelles in aqueous solution.^31^ When these components are in compatible concentrations, the membrane formed presents a structured and uniform distribution, which significantly reduces the likelihood of fractures due to the cohesion between the phases.

However, membranes classified as heterogeneous and brittle are represented by level 0 in Table 1 (e.g., membranes No. 3, 7, 11, and 15; Table S2, Figure 1b,c). In these membranes phase segregation was observed, which can be explained by the incompatibility of CT and Pluronic F127 concentrations. At higher concentrations of F127, for example, the formation of larger aggregates that are less integrated into the CT matrix is observed, leading to phase separation. This behavior reflects the tendency of Pluronic F127 to form self-associating structures that, when not sufficiently stabilized by the CT polymer matrix, result in areas of weakness. These segregated areas compromise membrane continuity, resulting in stress points where fracture occurs.^32^ On the other hand, in balanced formulations, Pluronic F127 contributes to membrane flexibility and stability, but without exceeding the point at which segregation occurs. These results indicate that membrane homogeneity is intrinsically linked to the interaction between CT and Pluronic F127. The cohesive and resistant structure of homogeneous membranes is achieved only when concentrations are compatible, maximizing the interaction between the polymeric phases and minimizing the occurrence of segregated areas and points of weakness.

#### 3.1.3 Polarized Light Optical Microscopy (PLOM)

PLOM was used to investigate the crystallinity of the membranes, with analysis performed in three distinct regions to ensure optimal magnification and structural detail. This assay is essential for assessing membrane homogeneity, as the desired application requires the absence or minimization of crystals, ensuring a uniform and functional structure. In general, the amorphous nature of drugs incorporated into membranes offers advantages related to the solubility and bioavailability of the active ingredient.^33^ This characteristic is particularly important for drugs with low water solubility, such as SSD (0.34 mg/100 mL^34^), as it facilitates their dissolution and promotes more efficient release at the application site. The amorphous form of active ingredients has higher free energy compared to the crystalline form, resulting in superior solubility in biological fluids.^35^ Thus, amorphous nature can optimize drug absorption through the skin, improving therapeutic efficacy and wound healing. Furthermore, this characteristic can be exploited to control prolonged or localized drug release, meeting specific wound treatment needs.

PLOM allows us to differentiate crystalline areas from amorphous regions in the polymer matrix. Light regions represent points of birefringence, indicating the presence of crystals, while dark areas reflect an amorphous and disorganized phase, as shown in Figure 2.

**Figure 2:**
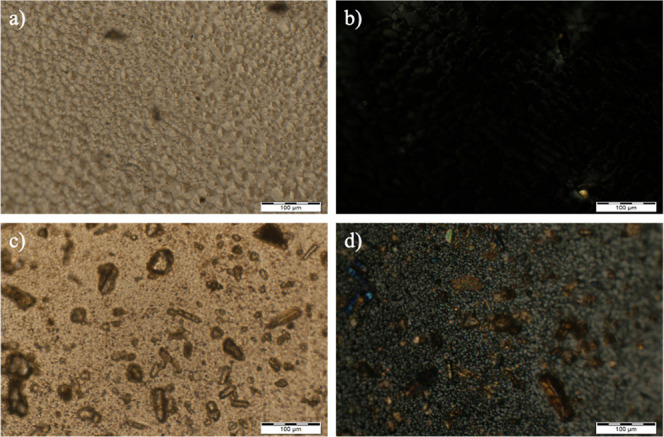
Optical microscopy of membranes No. 2 and No. 9. (a,b) Polarized light interference image. (c,d) Image under total polarization condition.

As mentioned above, the presence of crystals in membranes is undesirable, as it compromises uniform drug permeation by introducing structural barriers into the polymer matrix. Crystals can reduce drug release efficiency, hindering homogeneous diffusion and, consequently, limiting the therapeutic efficacy of cutaneous application.^36^ Therefore, ensuring the absence of crystallinity is essential for the development of an ideal membrane. The samples shown in Figure 2, in subfigures (a,c), correspond, respectively, to membrane No. 2, while subfigures (b,d) represent membrane No. 9.

After analyzing the 19 membranes obtained in the factorial design, crystallinity was categorized into three levels: 0 for crystals <10 μm, 1 for crystals between 10–100 μm, and 2 for crystals >100 μm. Most membranes presented crystals smaller than 10 μm, suggesting a more uniform dispersion of the crystals, which minimizes the direct impact on mechanical properties and contributes to the uniformity of the polymer matrix. However, some membranes (1, 5, 6, 9, 10, 12, 13, 14, 16, 17, 18, and 19) exhibited both small (<10 μm) and larger (>10 μm) crystals, which may indicate segregation and disorder in the polymer structure.

The presence of crystals larger than 10 μm introduces discontinuities in the matrix, favoring the formation of areas of weakness and compromising membrane homogeneity. These discontinuities create stress points that can weaken mechanical properties, impairing the uniform distribution of active components and, consequently, membrane efficacy.^37^ Therefore, the predominance of crystals <10 μm in the evaluated membranes suggests a more robust structure, while the occurrence of crystals >10 μm highlights the importance of strictly controlling the formulation process to avoid agglomerations that could compromise structural integrity. Therefore, the absence or minimization of crystallinity is essential to preserve mechanical integrity and ensure effective drug incorporation, certifying efficient and stable membrane application in the desired context.

#### 3.1.4 Simultaneous Analysis of the Responses

Before presenting the global optimization, three-dimensional surface plots were generated to illustrate how the combined variation of the formulation factors ([CT], [F127], [SSD], and [PIP]) influences the overall desirability (Figure 3a–f).

**Figure 3:**
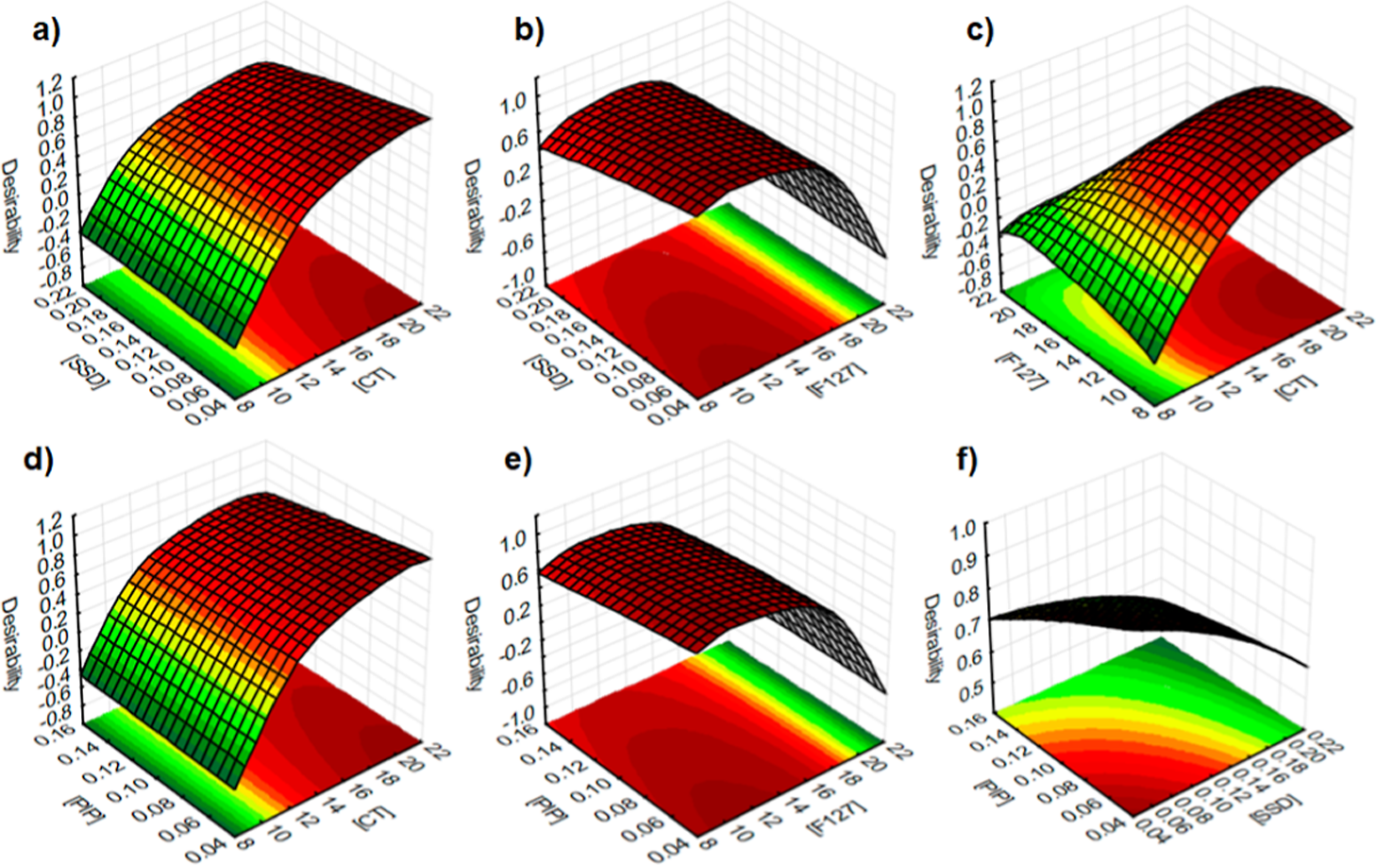
Three-dimensional response surface plots showing the influence of formulation factors on the overall desirability. (a) [SSD] vs [CT]; (b) [SSD] vs [F127]; (c) [CT] vs [F127]; (d) [PIP] vs [CT]; (e) [PIP] vs [F127]; and (f) [PIP] vs [SSD]. Green-to-red gradients represent increasing to decreasing desirability levels, respectively.

As shown in Figure 3a–f, the response surface plots illustrate the combined influence of the formulation factors on desirability. A strong synergistic effect between [CT] and [F127] is evident (Figure 3c), were increasing concentrations of CT markedly improved desirability. In contrast, [SSD] showed only a modest contribution when combined either with [CT] or [F127] (Figure 3a,b), suggesting a secondary role in the optimization. Regarding [PIP], a positive influence was observed when combined with [CT] (Figure 3d), but its interaction with [F127] or [SSD] (Figure 3e,f) produced limited gains.

After individually analyzing each of the responses used in the factorial design (membrane weight, thickness, uniformity/presence of defects and/or cracks, and sample crystallinity level), a simultaneous analysis of the responses was performed using the desirability function proposed by Derringer & Suich.^21^ The best conditions are presented in Figure 4.

**Figure 4:**
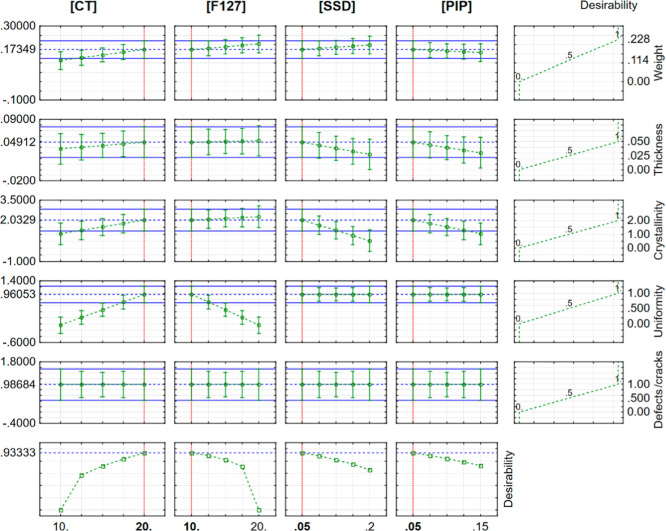
Individual desirability for each variable and the global condition.

The desirability function is a statistical tool used to optimize formulations in which multiple responses or variables need to be evaluated simultaneously. These values allow different responses (or factors) to be combined into a single numerical value, facilitating multidimensional analysis and identifying the ideal conditions for the system under study.^21^

In Figure 4, the global desirability was obtained by maximizing CT concentration and minimizing the other factors. The optimal condition (20 mg·mL^–1^ CT, 10 mg·mL^–1^ F127, 0.050 mg·mL^–1^ SSD, and 0.050 mg·mL^–1^ PIP) yielded the highest desirability index (DI = 0.93) and was selected for further studies. From now on, this sample will be referred to as CT/F127/PIP/SSD and its controls, following the concentrations defined in the planning, will be obtained: CT/F127, CT/F127/SSD, and CT/F127/PIP.

### 3.2 Physicochemical and Structural Characterization of Membranes

#### 3.2.1 Thermogravimetric Analysis (TGA)

TGA of the different membrane and component samples was performed to assess thermal stability and decomposition processes. Figure 5a,b shows the mass variation as a function of temperature for samples CT, F127, CT/F127, CT/F127/PIP, CT/F127/SSD, and the optimized complete membrane, allowing for a comparative analysis between the different formulations.

**Figure 5:**
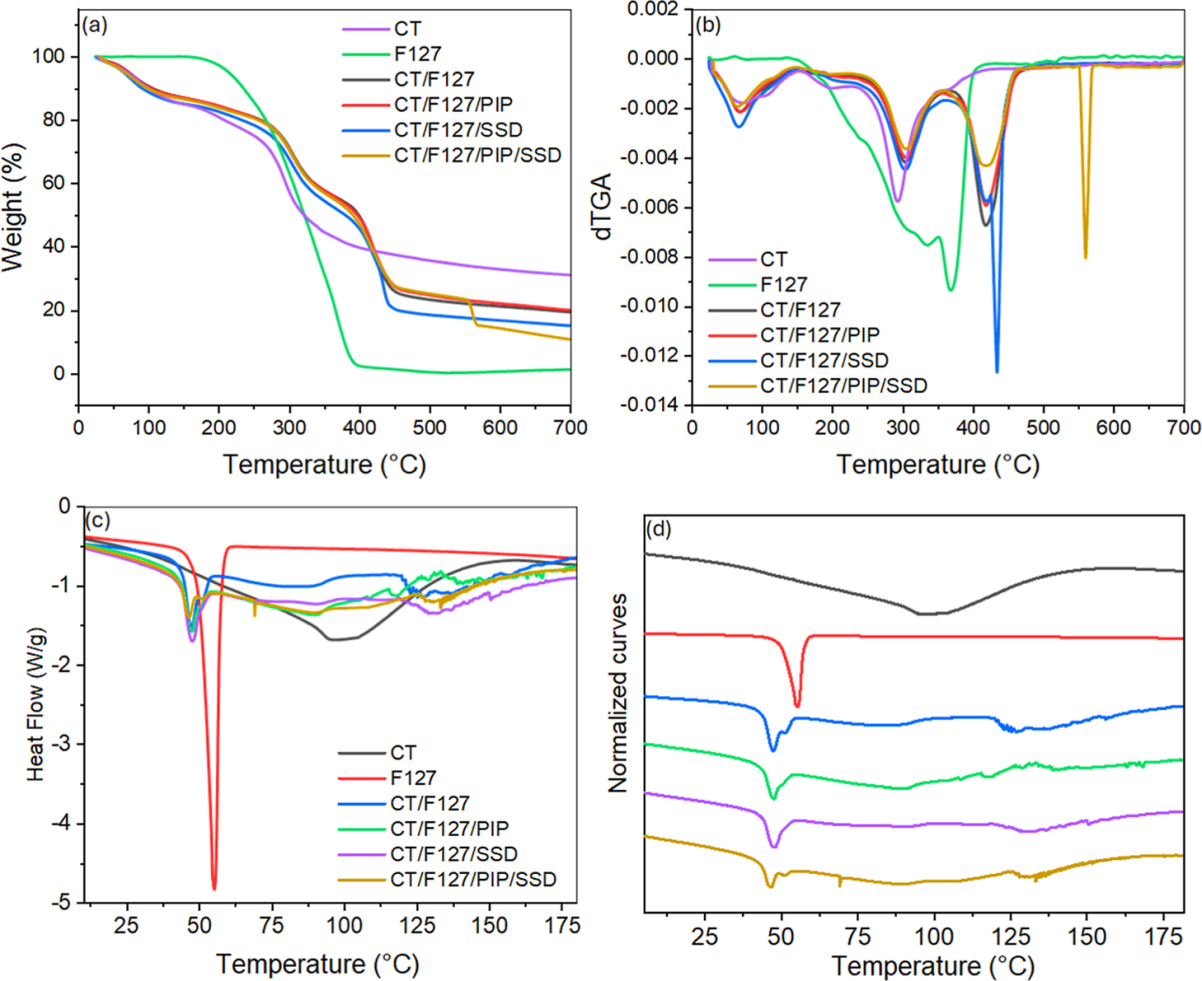
(a) TGA, (b) derivative thermogravimetry (DTG), (c) DSC curves, and (d) normalized DSC curves of the optimized membrane and its controls.

The first stage of mass loss, observed between 50 and 150 °C, is attributed to the evaporation of adsorbed or bound water. All samples exhibit a slight decrease in mass within this range, suggesting the presence of residual moisture likely absorbed during membrane preparation or storage. After the initial water loss, distinct thermal degradation bands are observed for each sample, particularly in the range between 200 and 400 °C. This second phase of mass loss is typically associated with the decomposition of the sample’s organic components, which exhibit different degradation onset temperatures reflecting their thermal stabilities. The CT sample, for example, begins to lose mass significantly earlier than the others due to its decomposition, indicating lower thermal stability compared to the other formulations.^38^ In contrast, the sample containing only the block copolymer exhibits a marked mass loss around 300–400 °C, which is consistent with the thermal decomposition of Pluronic F127.^39^

It is also observed that the CT/F127/SSD sample exhibits a slightly different degradation profile, with significant mass loss occurring in a slightly higher temperature range compared to the samples containing only CT or F127. This suggests that the presence of SSD in the formulation may confer a slight improvement on the membrane’s thermal stability, possibly due to interactions that stabilize the polymer matrix.

Above 600 °C, little to no additional mass loss is observed, indicating the near-complete degradation of organic content. The remaining mass, or residue, represents the thermally resistant fraction of the sample, as summarized in Table 2.

**Table 2:** Percentage of Residue Generated after Analysis (TGA)

sample	residue (%)
CT	31.1
F127	1.2
CT/F127	19.2
CT/F127/PIP	20.2
CT/F127/SSD	15.2
CT/F127/PIP/SSD	10.9

Among the samples analyzed, CT, CT/F127, and CT/F127/PIP presented the highest residual masses. This result may be related to the chemical composition of these samples, especially the higher proportion of CT, which, despite not being thermally stable, tends to yield a significant char residue upon decomposition due to partial carbonization of its structure.^40^

In contrast, when analyzing the CT membranes with additives, it is evident that the combination of PIP and SSD in CT/F127/PIP/SSD led to a reduction in residual mass. This suggests that the incorporation of both components alters the decomposition pathway, possibly promoting more complete thermal degradation. Therefore, the TGA results indicate that while CT contributes to higher char formation, the presence of SSD and PIP modulates the thermal degradation profile, with the optimized CT/F127/PIP/SSD membrane exhibiting greater thermal resistance, as seen by its delayed mass loss onset and sharper degradation peaks, compared to chitosan alone.

#### 3.2.2 Differential Scanning Calorimetry (DSC)

The results presented in Figure 5c,d correspond to the DSC analysis performed to characterize the membranes in terms of their thermal properties, stability, and possible interactions between components, essential factors for therapeutic applications. It was observed that the formulation containing only F127 was unable to form a structured membrane due to the absence of components that promote cohesion. As a result, this material had to be scraped and analyzed in powder form to enable thermal evaluation.

The CT membrane presented an endothermic peak between 50 and 150 °C which can be attributed to the evaporation of physically adsorbed water and the release of hydrogen-bonded water molecules within the polymer matrix.

F127 showed an endothermic peak at 55.2 °C, related to the melting of the copolymer.^41^ Samples containing CT in combination with F127 and other additives showed changes in their thermal profiles, indicating interactions between the components. For example, the CT/F127 membrane exhibited peaks at 47.2 and 87.6 °C, with the first attributed to F127 and the second to CT water loss. Similarly, in the CT/F127/SSD formulation, peaks were observed at 47.3 and 91.4 °C.

For the complete composition containing CT/F127/PIP/SSD, the thermogram revealed characteristic thermal behavior. Main peaks were identified at 46.1 and 51.7 °C, indicating transitions related to F127 and its interaction with the other components. These data reinforce the interaction between the matrix polymers and actives. Besides that, the results of the FTIR analysis of the samples can be found in the Supporting Information.

#### 3.2.3 Contact Angle Analysis

Contact angle measurements were performed to evaluate the wettability and surface hydrophilicity of the developed membranes. Table 3 summarizes the contact angles obtained for each sample, and representative images are shown in Figure 6.

**Table 3:** Summary of the Physicochemical and Mechanical Properties of the Membranes^a^

samples	contact angle (°) mean ± SD	roughness (nm)	dissolution degree (%)	fracture stress (MPa)	fracture strain (%)	Young’s modulus (MPa)
CT	86.5 ± 12.8	10	29.8			
CT/F127	51.7 ± 10.7	13	35.1	19.1 ± 6.3	21.89 ± 9.1	959.5 ± 198.7
CT/F127/PIP	63.2 ± 15.2	67	37.3	19.6 ± 3.5	11.6 ± 3.8	1045.1 ± 486.1
CT/F127/SSD	61.8 ± 2.0	16	43.3	24.0 ± 10.9	10.8 ± 3.4	1282.5 ± 687.3
CT/F127/PIP/SSD	72.3 ± 2.3	56	38.7	25.1 ± 2.2	6.6 ± 1.7	1552.2 ± 308.7

a Static contact angles for water droplets on membrane surfaces, surface roughness values determined by AFM using WSxM 5.0 software, percentage of membrane dissolution in phosphate buffer (pH 7.4, 37 °C) after 24 h, and mechanical properties, including fracture stress, fracture strain, and Young’s modulus. All values are presented as mean ± standard deviation for each sample composition: CT/F127, CT/F127/PIP, CT/F127/SSD, and CT/F127/PIP/SSD.

**Figure 6:**
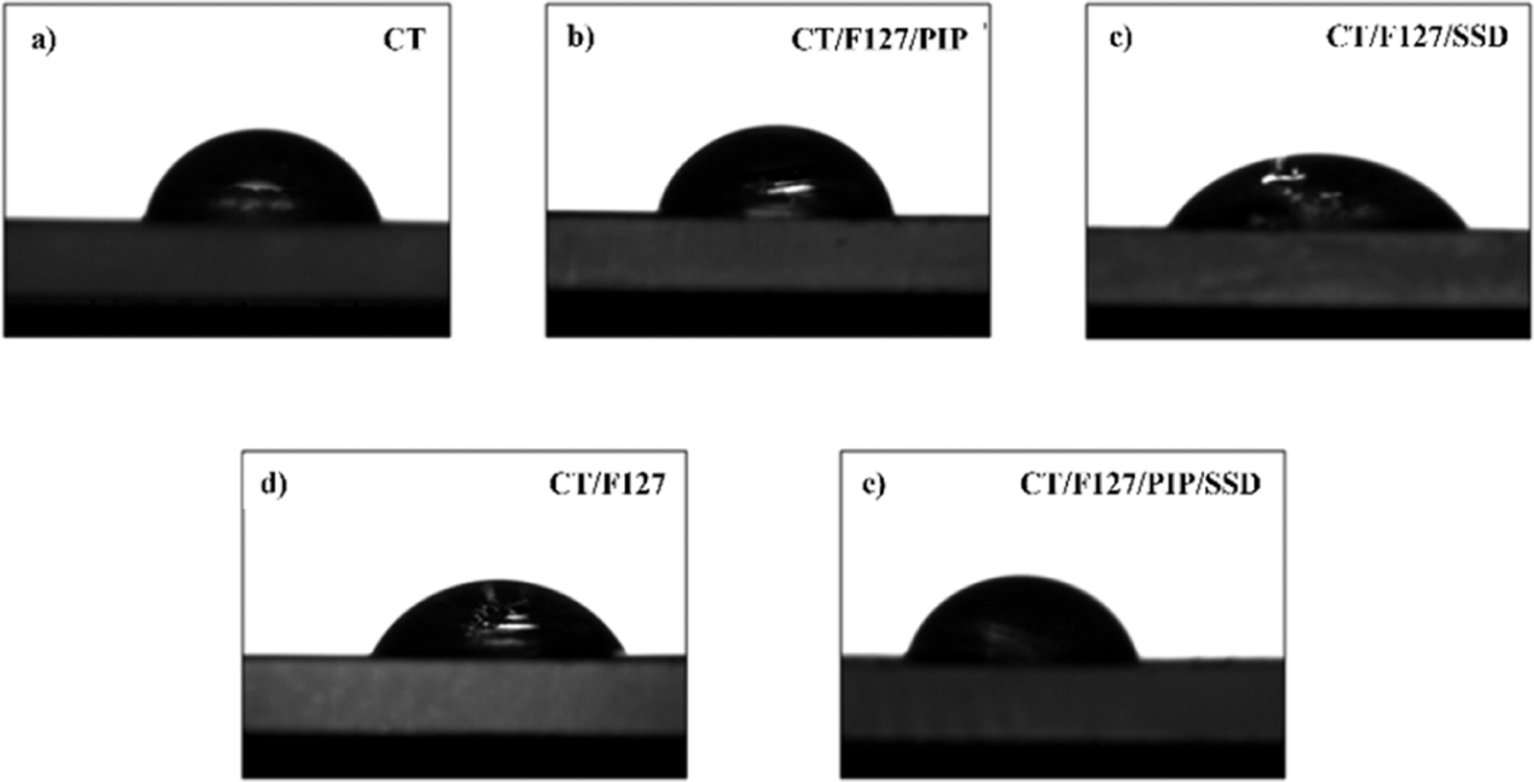
Representative images of water droplets on the surface of the different membranes during contact angle analysis: (a) CT, (b) CT/F127/PIP, (c) CT/F127/SSD, (d) CT/F127, and (e) CT/F127/PIP/SSD. The images illustrate the differences in surface wettability among the membranes, with variations in droplet shape indicating changes in hydrophilicity/hydrophobicity due to the incorporation of PIP and SSD.

The CT membrane alone presented the highest contact angle (86.5°), confirming its more hydrophobic surface. In contrast, the sample composed of CT/F127 exhibited the lowest average contact angle (51.7°), indicating a hydrophilic surface which can be attributed to the presence of Pluronic F127, known for its affinity for water due to the polyoxyethylene units in its structure. Upon incorporation of PIP into the CT/F127/PIP sample resulted in an increased average contact angle (63.2°), suggesting a moderate reduction in surface hydrophilicity. This effect is likely due to the hydrophobic character of PIP (log *P* ≈ 2.78),^42^ which may have reduced surface interaction with water by introducing hydrophobic characteristics to the matrix. The CT/F127/SSD membrane exhibited a similar contact angle (61.8°), reflecting the amphiphilic nature of SSD (log *P* ≈ 2.1).^43^ Finally, the optimized membrane CT/F127/PIP/SSD displayed an intermediate contact angle of 72.3°. This result reflects a cumulative effect of the components, where F127 contributes to hydrophilicity, while PIP and SSD modulate surface properties toward a more hydrophobic profile. This value aligns closely with the range (40°–70°) recognized as most effective for cellular attachment, providing an excellent balance of protein adsorption and cell–surface interactions.^44^

#### 3.2.4 Atomic Force Microscopy (AFM)

Analyses performed using AFM allowed the characterization of the surface morphology of the developed membranes, highlighting the influence of the different components incorporated into the matrix. Roughness, which refers to the variation in surface height at different scales, is a crucial parameter for assessing the quality and functionality of membranes, especially in cutaneous applications, as it can affect cell adhesion, drug release, and interaction with the skin.^44^ The 2D and 3D images of the membranes are shown in Figure 7, while the roughness values are presented in Table 3.

**Figure 7:**
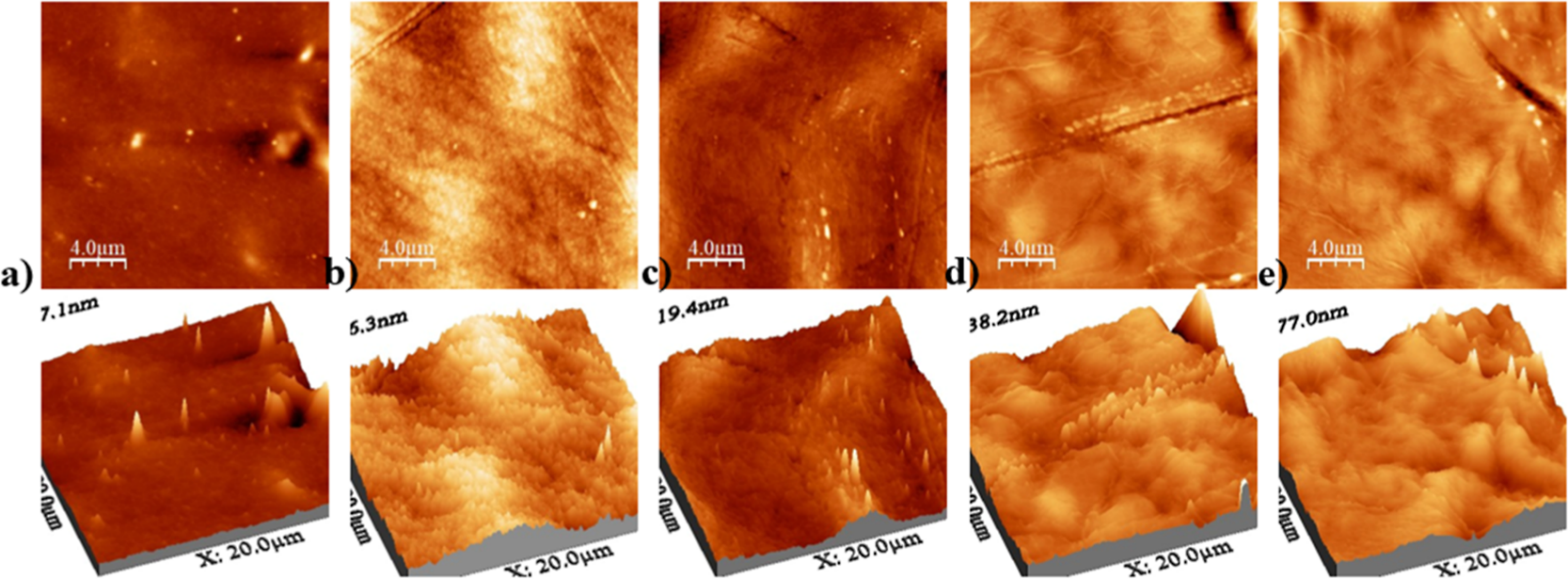
2D and 3D AFM images of membranes: (a) CT, (b) CT/F127, (c) CT/F127/SSD, (d) CT/F127/PIP, (e) CT/F127/PIP/SSD.

The CT membrane displayed a relatively smooth surface, with an average roughness of 10 nm, consistent with the homogeneous structure typically formed by CT-based matrices.^45^ The addition of Pluronic F127 (CT/F127) slightly increased the roughness to 13 nm, likely due to polymer–polymer interactions that introduce minimal surface heterogeneity while preserving overall matrix uniformity.

Incorporation of SSD into the CT/F127 matrix (CT/F127/SSD) led to a moderate increase in roughness to 16 nm. This change may be attributed to the dispersion of SSD within the matrix and the formation of a more textured surface, consistent with its partial miscibility and interaction with the polymeric network. A more pronounced change was observed upon inclusion of PIP (CT/F127/PIP), which caused a substantial increase in surface roughness to 67 nm. This increase suggests poor dispersion and potential aggregation of PIP within the matrix, consistent with its low polarity and reduced compatibility with the hydrophilic polymer components. Linking the AFM results with the contact angle to the set CT/F127/PIP/SSD where was found higher contact angle (72°) was found, this suggests that the interaction between PIP/SSD is similar to SSD/SSD, facilitating a better accommodation of molecules, indicating an improvement in drug release and skin permeation.

Interestingly, the optimized formulation containing all components (CT/F127/PIP/SSD) exhibited a reduced roughness of 56 nm compared to CT/F127/PIP. This decrease suggests that the presence of SSD, along with the structural influence of F127 and CT, contributes to a partial smoothing of the surface, likely by mitigating PIP aggregation and promoting a more balanced dispersion of components.

Furthermore, in all formulations the AFM images showed preservation of the membrane’s characteristic striations, indicating that the matrix architecture remained intact despite the incorporation of the actives. These results suggest that membrane roughness can be effectively modulated through the strategic combination of functional components, allowing surface characteristics to be tuned for specific biomedical applications. It is important to note that in all cases, the roughness stayed far below 100 nm, a threshold reported in the literature as potentially limiting bacterial adhesion and proliferation.^46^

#### 3.2.5 Determination of Membrane Dissolution Rate

The dissolution behavior of the optimized membranes was evaluated under physiologically relevant conditions to assess their stability and disintegration profiles. As shown in Table 3, all membranes exposed to the acidic buffer (pH 5.5) underwent complete dissolution within 24 h, indicating high sensitivity to mildly acidic environments. This behavior may be advantageous for the treatment of acute wounds, as the healing process is accompanied by a gradual shift toward a lower pH.^47^ In such conditions, accelerated membrane disintegration could facilitate rapid drug release, contributing to a more targeted and localized therapeutic effect.

In contrast, when incubated at pH 7.4, the membranes displayed partial dissolution, with values ranging from ∼30% to 43%. This behavior can be explained by the fact that at pH 7.4 CT is above its p*K*_a_ (∼6.3), resulting in mostly deprotonated amino groups. Under these conditions, nearly 90% of the amine groups are uncharged, significantly reducing CT solubility. This implies improved structural stability at near-neutral pH, akin to healthy skin, and supports the potential for controlled and sustained drug release under normal physiological conditions.

This pH-responsive dissolution behavior is particularly relevant for wound treatment. Chronic or infected wounds often present an elevated pH (≥8), frequently associated with bacterial colonization (e.g., *P. aeruginosa*) and delayed healing. In such alkaline environments, fibroblast and keratinocyte function is impaired, and the activity of enzymes involved in tissue regeneration is diminished.^48^ The reduced dissolution rate observed at pH 7.4 suggests that these membranes could remain stable for extended periods, allowing sustained drug release while inhibiting microbial growth and supporting pH normalization.

Together, these findings indicate that the membranes can be tailored to respond to the skin’s microenvironmental pH, enabling faster disintegration under acidic conditions and prolonged stability under neutral or alkaline conditions. This dual behavior makes them promising candidates for pH-responsive drug delivery in dermatological applications.

#### 3.2.6 Determination of Membrane Swelling Degree

Membrane swelling degree analysis assess their water absorption capacity, an essential property for wound dressings, as it allows for exudate management and promotes a moist healing environment. The procedure consisted of immersing membrane samples in a phosphate buffer solution (pH 7.4) at 37 °C, simulating physiological conditions. The samples were weighed at regular intervals, and the degree of swelling was calculated based on the change in mass over time, as shown in Figure 8.

**Figure 8:**
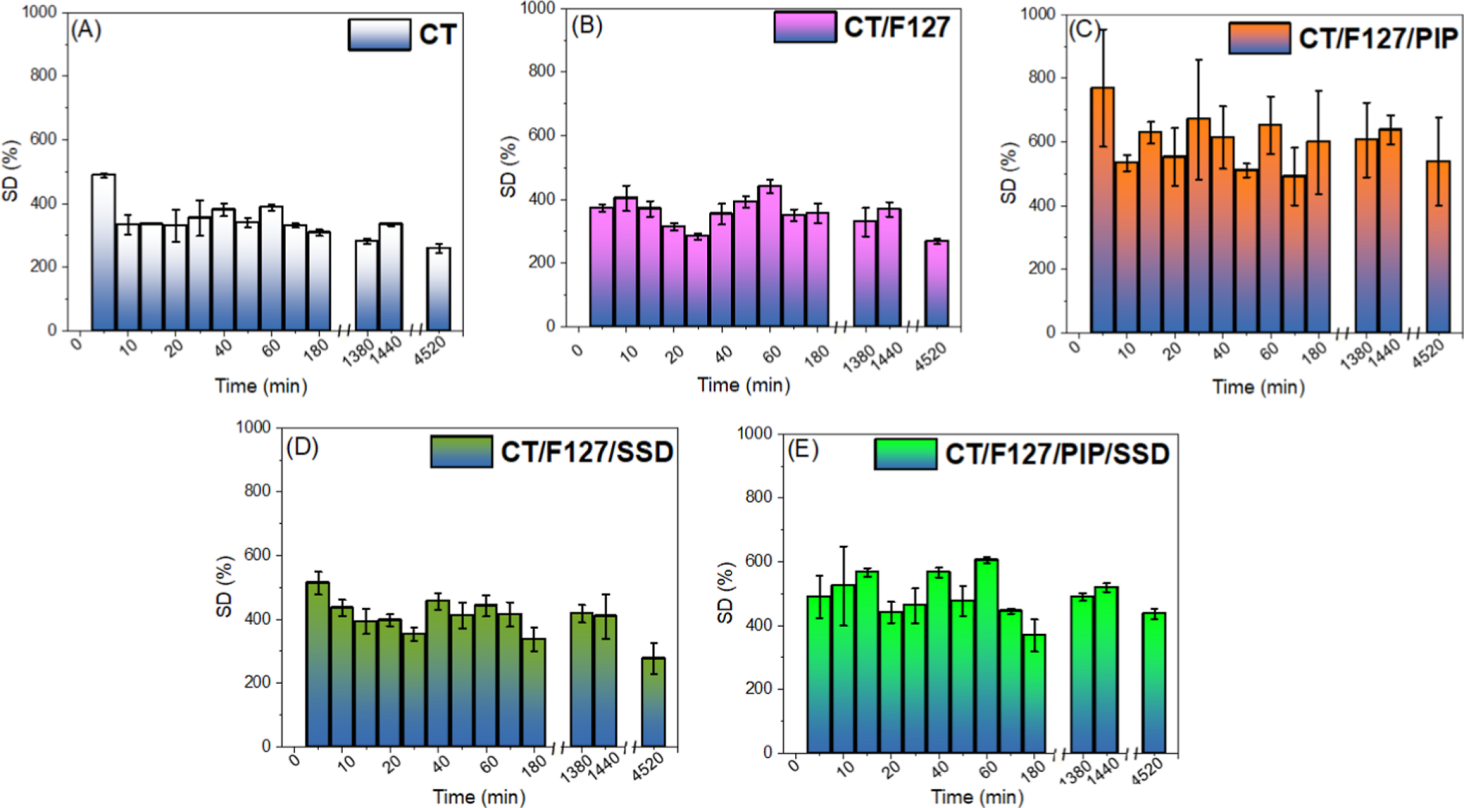
Swelling degree (SD) of the different formulations as a function of time at pH 7.4 at 37 °C. (a) CT, (b) CT/F127, (c) CT/F127/PIP, (d) CT/F127/SDD, (e) CT/F127/PIP/SSD.

The results showed that the membranes rapidly absorb large volumes of water, reaching a plateau in the short term, indicating saturation. The CT membrane had the lowest swelling capacity, while the blended membranes (CT/F127, CT/F127/PIP, CT/F127/SSD, and CT/F127/PIP/SSD) demonstrated better performance. The addition of F127 conferred greater absorption, while SSD, although slightly reducing swelling capacity, provided antimicrobial properties. The CT/F127/PIP/SSD combination exhibited the most balanced profile, with high absorption and prolonged stability, making it the most promising for multifunctional dressings.

The results of this study, which demonstrated increased swelling of membranes, are consistent with other studies that indicated greater water absorption in CT membranes combined with F127.^49^ The addition of SSD did not result in significant effect on the swelling degree. The optimized membrane CT/F127/PIP/SSD, however, performed well, balancing absorption and stability.

It is noteworthy that under acidic conditions (acetate buffer, pH 5.5), all membranes dissolved within 24 h (as reported previously), which may limit their direct application in environments with low pH. For such use cases, further cross-linking or compositional adjustments may be required to ensure structural integrity and therapeutic performance. Overall, the swelling results support the application of these membranes for exudate absorption and wound healing, particularly in environments close to physiological or slightly alkaline pH, such as chronic wounds or nonhealing ulcers.

#### 3.2.7 Mechanical Properties

The mechanical behavior of the CT/F127-based membranes, presented in Figure 9 and detailed in Table 3, demonstrates the distinct effects of incorporating PIP and SSD on membrane performance. The control membrane (CT/F127) displayed the lowest tensile strength (∼1.0 kg/mm^2^) but the highest elongation (∼22%), confirming its superior flexibility but limited mechanical resistance. The CT/F127/PIP membrane demonstrated moderate tensile strength (∼1.6 kg/mm^2^) and greater strain at break (∼15%), suggesting that PIP contributed to improved elasticity without substantially reinforcing the structure. The CT/F127/SSD formulation exhibited the highest tensile strength (∼3.6 kg/mm^2^) but also the lowest elongation at break (∼12%), indicating that the addition of SSD substantially increased membrane rigidity while compromising flexibility. This behavior can also be explained by the partial crystallinity introduced by SSD within the membranes. The crystalline domains act as rigid reinforcement sites, enhancing tensile strength by restricting polymer chain mobility. However, this restriction reduces the capacity for plastic deformation, which accounts for the significant loss of elongation and the consequent brittle character of the SSD-containing membranes.

**Figure 9:**
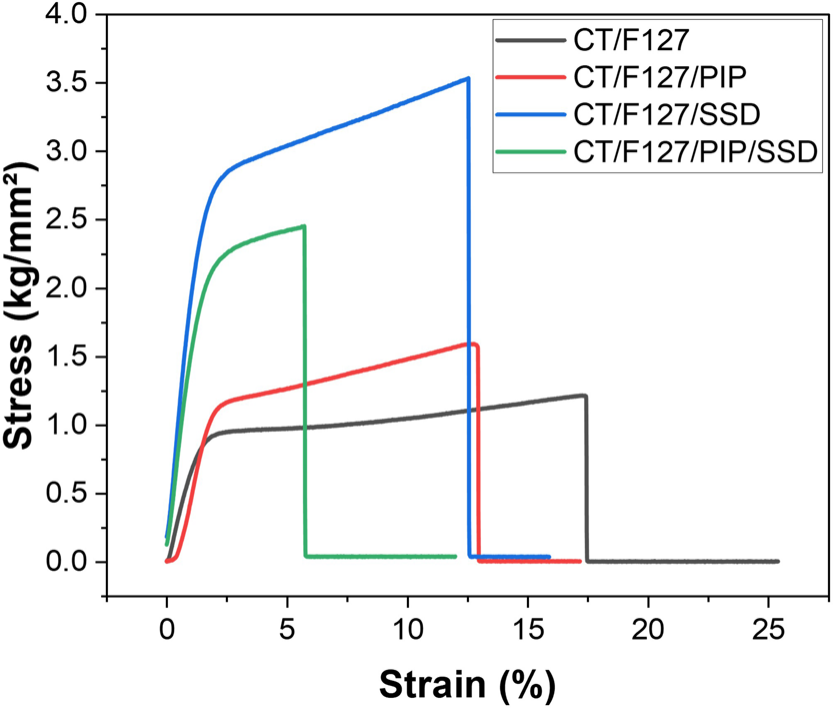
Stress–strain curves of chitosan/F127-based membranes with and without the incorporation of PIP and SSD.

The combined formulation, CT/F127/PIP/SSD, presented an intermediate strength (∼2.3 kg/mm^2^) but the lowest elongation (∼6%), implying a combined stiffening effect that led to greater brittleness. Overall, SSD enhanced strength at the expense of flexibility, PIP provided moderate improvements in elasticity, and the dual-loaded membrane balanced both properties but with increased brittleness.

The stress–strain curves of hydrated chitosan/F127-based membranes, with and without PIP and SSD, are shown in Figure S2. However, the 500 N load cell used in our setup was not sensitive enough for the low-force range of these thin, flexible membranes, resulting in noisy stress–strain data. In general, hydrated membranes (Figure S2) appeared to behave differently from their dry counterparts (Figure 9), tending to be more flexible and deformable, with a possible increase in elongation at break. This may result from disruption of polymer–polymer interactions, such as hydrogen bonding. Such behavior could better reflect the membranes’ performance under physiological conditions, where they would contact wound exudates, suggesting their potential suitability as wound dressings.

### 3.3 Ex Vivo Porcine Skin Drug Deposition Studies

Ex vivo drug deposition studies were conducted using two membrane formulations (CT/F127/SSD and CT/F127/PIP/SSD) and two control suspensions (SSD in propylene glycol, PG, and PIP/SSD in PG), enabling a comparative evaluation of permeation profiles across porcine ear skin, as presented in Table 4.

**Table 4:** Ex Vivo Quantification of SSD Deposition in the Epidermis and Dermis of Porcine Ear Skin

sample	Ag° in epidermis (ug g^–1^)	Ag° in dermis (ug g^–1^)
SSD/PG	44.90 ± 19.36	8.35 ± 2.71
PIP/SSD/PG	44.23 ± 19.46	5.40 ± 0.99
CT/F127/SSD	9.82 ± 2.12	2.24 ± 1.46
CT/F127/PIP/SSD	21.75 ± 6.45	4.99 ± 0.54

As shown in Table 4, incorporating PIP into the membrane more than doubled SSD concentration in the epidermis (from 9.82 to 21.75 μg g^–1^) and dermis (from 2.24 to 4.99 μg g^–1^). This significant enhancement demonstrates that PIP plays a key role in promoting SSD permeation through the skin layers, confirming the effectiveness of the membrane as a delivery platform. This retention may also be related to the presence of F127, which has been reported to favor the deposition of poorly soluble drugs by interacting with skin layers, especially when combined with CT.^50^

Although control samples (SSD/PG and SSD/PIP/PG) exhibited higher absolute permeation values in the epidermis and dermis compared to membrane systems, it is important to note that the standard deviations in PG dispersions are much higher than those observed in the membrane systems, indicating poor reproducibility and inconsistent drug delivery. Moreover, the addition of PIP in suspension did not produce a meaningful improvement over PG alone. In this context, PG acts only as a control solvent, while the membrane matrix provides the real advantage by significantly enhancing SSD permeation in the presence of PIP. Another relevant point is that the comparison between the best dermis results for the control (8.35 ± 2.71 μg g^–1^, *n* = 3) and for the membrane (4.99 ± 0.54 μg g^–1^, *n* = 3) does not indicate a statistically significant difference, considering the standard deviations and sample size. This suggests that the advantage of the membranes lies less in achieving higher absolute deposition values and more in ensuring reproducibility, consistent dosing, and practical applicability.

Besides that, these dispersions in PG are not practical for burn wound application due to their high fluidity, which reduces residence time on irregular wound surfaces. This limitation may lead to suboptimal dosing and patient discomfort, issues previously reported for commercial SSD creams.^13^ In addition, recent studies have indicated that such formulations may cause skin sensitization and allergic contact dermatitis.^51^

In contrast, the membrane-based systems demonstrated superior performance by enabling prolonged SSD retention in both dermis and epidermis, ensuring sustained release and extended coverage of the skin surface. The combination of CT, F127, and PIP not only improved SSD permeation but also addressed the practical limitations of liquid formulations, offering controlled dosing, reduced application frequency, greater patient compliance, and lower risks of systemic toxicity.

### 3.4 In Vitro Bactericidal Assay in ATCC and Clinical Strain Lineages

Microdilution experiments in plates were carried out to evaluate the effectiveness of membranes containing SSD and PIP against both clinical and ATCC *E. coli* and *S. aureus* strains. We begin by presenting the results for *E. coli* ATCC.

The complete membrane exhibited the highest inhibitory effect against *E. coli* ATCC (Figure 10A), with approximately 50% reduction in bacterial viability after 24 h. Among the tested membranes, only those containing SSD showed a statistically significant difference compared to the negative control. Furthermore, when comparing the membrane containing PIP with the one incorporating both PIP and SSD, a statistically significant difference was also observed, highlighting the crucial role of SSD in the antimicrobial activity of the membranes. In addition, the positive control (disk containing 25 μg of SUL) led to a reduction of approximately 25% in bacterial viability, indicating an intrinsic resistance of this strain to SSD. Notably, the incorporation of SSD into the membranes, especially in combination with PIP, resulted in a greater reduction in viability, which may be associated with enhanced release and absorption of SSD in the presence of PIP.

**Figure 10:**
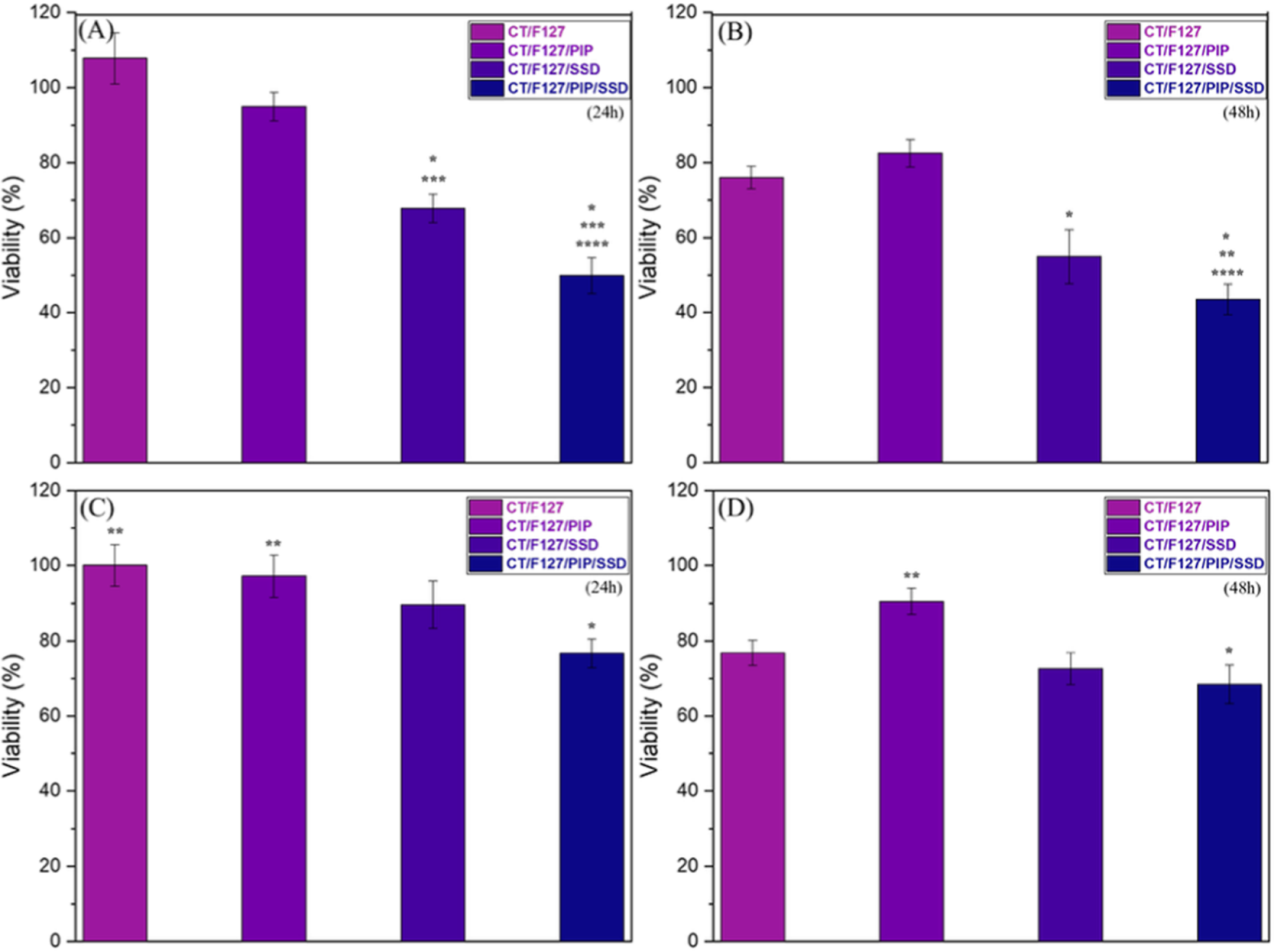
Viability of *E. coli* ATCC after treatment with the complete membrane and control systems at (a) 24 h and (b) 48 h, and viability of *S. aureus* ATCC under the same conditions at (c) 24 h and (d) 48 h. Statistical significance (*p* < 0.05): (*) vs control; (**) vs SUL disc; (***) vs CT/F127; (****) vs CT/F127/PIP; (*****) vs CT/F127/SSD.

After the second application and viability analysis at 48 h (Figure 10B), a further decrease in bacterial viability (55%) was observed, suggesting that prolonged exposure to the drug enhances its effect. However, no statistically significant differences were detected between the 24 and 48 h applications.

For the *S. aureus* ATCC strain (Figure 10C,D), the greatest reduction in bacterial viability was observed with the complete membrane, around 24% after 24 h (Figure 10C), being the only condition that showed a statistically significant difference compared to the control. The reduced effect against *S. aureus* may be related to the thick peptidoglycan layer characteristic of Gram-positive bacteria, which hinders drug penetration and consequently decreases treatment efficacy. After the second application and evaluation at 48 h (Figure 10D), a further reduction in viability was observed with the complete membrane, which again was the only condition significantly different from the control. However, no statistical differences were detected between the 24 and 48 h time points.

Given the results obtained with the ATCC strains, we proceeded to the evaluation of the clinical strains. Unlike reference strains, which display a more stable and standardized susceptibility profile, clinical strains may exhibit greater phenotypic variability and develop multiple resistance mechanisms. Therefore, an initial susceptibility test (SUL) was performed to characterize the response of these strains to the proposed treatments. For this the disk-diffusion method was applied, with the aim of determining whether the strains presented resistance, intermediate susceptibility, or susceptibility to the drug. According to BrCAST, for both strains, susceptibility to SUL is considered for halos up to 2 cm, intermediate susceptibility for halos between 2.1 and 4 cm, and resistance for halos larger than 4 cm.^52^ Thus, *E. coli* presented a halo of 2.6 cm and *S. aureus* of 3.2 cm, indicating that both clinical strains show intermediate resistance to SUL, that is, they are susceptible only when exposed to concentrations higher than the usual therapeutic doses.

Based on these susceptibility results, we started the microdilution experiments in plates. In these tests, for *E. coli* (Figure 11A), the complete membrane (CT/F127/SSD/PIP) showed the highest inhibition, with about 76% reduction in bacterial viability after 24 h. In contrast, the membrane containing only SSD promoted a 33% reduction, indicating that the incorporation of PIP enhanced the antimicrobial effect of SSD. It is important to highlight that PIP control did not differ statistically from the negative control (CT/F127), suggesting that, at this concentration, PIP does not act directly as a bactericidal agent, but contributes to increasing the efficacy of SSD, possibly by modulating resistance mechanisms.

**Figure 11:**
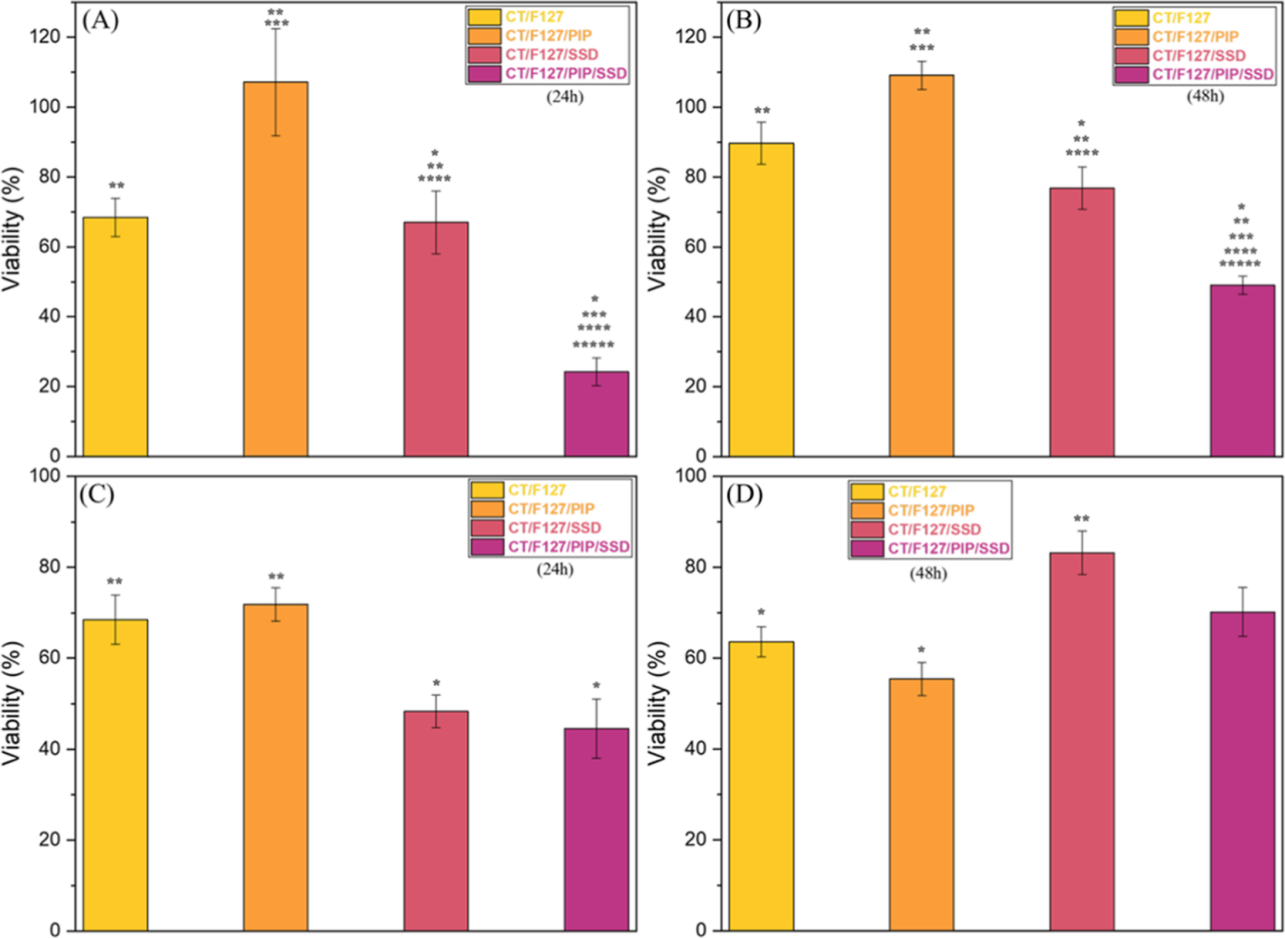
Viability of *E. coli* clinical strain after treatment with the complete membrane and control systems at (a) 24 h and (b) 48 h, and viability of *S. aureus* clinical strain under the same conditions at (c) 24 h and (d) 48 h. Statistical significance (*p* < 0.05): (*) vs control; (**) vs SUL disc; (***) vs CT/F127; (****) vs CT/F127/PIP; (*****) vs CT/F127/SSD.

Indeed, the literature reports that PIP acts as an inhibitor of the efflux pump that reduces the intracellular concentration of antibiotics. Khan et al. used ethidium bromide (EtBr) as a fluorescent probe to observe the effect of PIP on this mechanism.^53^ When present inside the cell, EtBr intercalates with DNA and emits strong fluorescence, while in the extracellular medium the fluorescence is significantly lower. In the presence of PIP, increased fluorescence was observed, indicating that the efflux pump was inhibited. Thus, the results obtained in this study are consistent with this mechanism, since the addition of PIP to the membrane enhanced the action of SSD, resulting in greater reduction of bacterial viability (76% versus 33%).

This interpretation is reinforced by the controls used. The positive control, SUL discs, demonstrated that only the complete membrane did not show a statistically significant difference, suggesting that the presence of PIP contributes to increasing the diffusion and prolonged release of SSD. On the other hand, the membrane containing only PIP (CT/F127/PIP) showed lower antimicrobial effect compared to the blank membrane (CT/F127), indicating that, in isolation, PIP does not confer significant bactericidal activity under the studied conditions.

This absence of direct effect can be explained by two factors. First, the concentration of PIP present in the membranes is below the minimum inhibitory concentration (MIC) reported for *E. coli*, estimated at 12 mg/mL by Lokhande et al.^54^ Second, PIP may accumulate on the membrane surface, interfering with the electrostatic interaction between the positive charges of CT and the negative groups of the bacterial cell wall, an interaction responsible for the intrinsic antimicrobial effect of CT.^55^ Therefore, while PIP is not sufficient to directly inhibit bacterial growth, its presence modifies the polymeric matrix in a way that optimizes the release and effect of SSD, as presented in ex vivo porcine skin drug deposition studies.

When analyzing statistical significance for 24 h (Figure 11A), it was observed that only the membranes containing SSD showed a significant difference compared to the control (CT/F127), confirming that the main antimicrobial action is associated with SSD. When compared to the positive control (SUL disc), all membranes, except the complete one, presented a significant difference, reinforcing that the incorporation of PIP is crucial to improve SSD release and inhibit bacterial resistance mechanisms. Furthermore, the incorporation of SSD did not result in a significant difference compared to the blank membrane.

With the application of a second membrane and evaluation at 48 h (Figure 11B), an increase in viability was observed in all samples when compared with the first application in 24 h, which may be related to the intermediate susceptibility characteristic presented by *E. coli* in the susceptibility tests. A statistically significant difference was detected exclusively for the membranes containing SSD in comparison to the control. Furthermore, no significant differences were observed among the treatments at either 24 or 48 h.

Finally, the treatment against the clinical *S. aureus* strain was evaluated, Figure 11C,D. For this strain, the greatest decrease in cell viability was observed with the complete membrane, showing an approximate reduction of 56% after 24 h. However, the incorporation of PIP did not result in a significant difference when comparing membranes containing only SSD with those containing SSD and PIP. This outcome may be associated with the absence of resistance mechanisms in *S. aureus* that are effectively inhibited by PIP. As previously discussed, one of the main targets of PIP is the inhibition of efflux pumps; however, this is not a predominant resistance mechanism in *S. aureus*.^56^

This bacterial species is more commonly characterized by mechanisms such as alterations in the peptidoglycan cell wall and the production of inactivating enzymes, such as β-lactamase, which confer resistance to β-lactam antibiotics, as observed in methicillin-resistant strains (MRSA).^56^ Therefore, the lack of efficacy of PIP can be explained by the fact that efflux pumps do not constitute a relevant resistance mechanism for *S. aureus*.

Additionally, after the second application of the membrane, an increase in bacterial viability to approximately 48% was observed (Figure 11D), which may be related to the intermediate sensitivity of the clinical strain to SSD, as previously evidenced by inhibition halo assays.

## 4 Conclusions

The development of CT/Pluronic F127 membranes loaded with SSD and PIP demonstrated that the rational design of bioinspired polymeric systems can effectively combine antimicrobial activity, controlled drug delivery, and potential wound healing benefits. The optimized formulation demonstrated adequate mechanical stability, homogeneity, as well as high thermal stability and moderate hydrophilicity. The membranes exhibited remarkable water absorption and swelling capacity, providing a moist environment favorable for tissue regeneration. Ex vivo permeation studies confirmed that PIP significantly enhanced SSD deposition in both the epidermis and dermis, showing that incorporating PIP more than doubled SSD deposition in both the epidermis (9.82 → 21.75 μg g^–1^) and dermis (2.24 → 4.99 μg g^–1^). In vitro microbiological assays demonstrated potent antimicrobial activity against both standard and clinical strains of *E. coli* and *S. aureus*, with PIP further amplifying SSD’s bactericidal effect for *E. coli* clinical strains, as the bactericidal effect of SSD values from 76% vs 33% reduction, consistent with its reported role as an efflux pump inhibitor.

These findings reinforce the importance of integrating natural and synthetic components to overcome the limitations of conventional dressings, providing multifunctionality in a single platform. Beyond highlighting their applicability in burn wound management, this work underscores a broader contribution to the field of advanced biomaterials, paving the way for innovative strategies in skin regeneration. Future research should deepen the understanding of drug release kinetics and permeation mechanisms, while also expanding preclinical evaluations to bridge the gap toward clinical translation. These results indicate that the membranes may serve as a promising platform for wound healing applications, including burn care, while further in vivo studies, cytotoxicity assessments, and long-term stability evaluations are required to confirm their therapeutic potential.

## Supplementary Material



## Notes

Data of coding and decoding of factorial experimental design models; data of thickness and weight measurement for the obtained membranes; data of desirability indexes (DI) for bioinspired membranes; additional information on the Fourier transform infrared spectroscopy (FTIR) and stress–strain curves of chitosan/F127-based membranes with and without the incorporation of PIP and SSD after hydration (PDF)
