# Supplementary material for: Bioinspired Membranes with Silver Sulfadiazine and Piperine for Enhanced Cutaneous Permeability
Source: ACS Omega. 2025 Oct 29;10(44):53486–502. doi: 10.1021/acsomega.5c08994 (PMC12613115; doi:10.1021/acsomega.5c08994)
Supplement: Supplementary file 1 [file ao5c08994_si_001.pdf]

Supporting information

Bioinspired Membranes with Silver Sulfadiazine and Piperine for Enhanced Cutaneous Permeability

Gabriely Cristini Batista de Deus[1], Heloisa Diehl Doring[1], Yara Schuvinski Ricken[1], Marta Elisa Rosso Dotto[2], Diego Galvan[1], Tatiana Herrerias[3], Eloah Latocheski[1*], Camila Fabiano de Freitas[1*]

[1]Department of Chemistry, Federal University of Santa Catarina, Florianópolis – Santa Catarina, 88040-900, Brazil.
[2]Department of Physics, Federal University of Santa Catarina, Florianópolis – Santa Catarina, 88040-900, Brazil.
3 Department of Clinical Analysis, Federal University of Santa Catarina, Florianópolis, Santa Catarina, Brazil

*Corresponding authors:

Eloah Latocheski eloah.latocheski@posgrad.ufsc.br and Camila Fabiano de Freitas Marin camila.f.freitas@ufsc.br

Table S1. Coding and decoding of factorial experimental design models.

Exp.	[CT]	[F127]	[SSD]	[PIP]	[CT] (mg mL^{-1})	[F127] (mg mL^{-1})	[SSD] (mg mL^{-1})	[PIP] (mg mL^{-1})
1	-1	-1	-1	-1	10	10	0.050	0.050
2	1	-1	-1	-1	20	10	0.050	0.050
3	-1	1	-1	-1	10	20	0.050	0.050
4	1	1	-1	-1	20	20	0.050	0.050
5	-1	-1	1	-1	10	10	0.200	0.050
6	1	-1	1	-1	20	10	0.200	0.050
7	-1	1	1	-1	10	20	0.200	0.050
8	1	1	1	-1	20	20	0.200	0.050
9	-1	-1	-1	1	10	10	0.050	0.200
10	1	-1	-1	1	20	10	0.050	0.200
11	-1	1	-1	1	10	20	0.050	0.200
12	1	1	-1	1	20	20	0.050	0.200
13	-1	-1	1	1	10	10	0.200	0.200
14	1	-1	1	1	20	10	0.200	0.200
15	-1	1	1	1	10	20	0.200	0.200
16	1	1	1	1	20	20	0.200	0.200
17	0	0	0	0	15	15	0.125	0.125
18	0	0	0	0	15	15	0.125	0.125
19	0	0	0	0	15	15	0.125	0.125

Table S2. Thickness and weight measurement data for the obtained membranes.

Membrane	Weight (g)	Central point (mm)	Lateral end 1 (mm)	Lateral end 2 (mm)	Mean thickness (mm)	Mean deviation (mm)
1	0.114	0.034	0.025	0.033	0.0307	0.004
2	0.170	0.040	0.045	0.040	0.042	0.002
3	0.094	0.041	0.037	0.043	0.040	0.002
4	0.212	0.049	0.043	0.050	0.047	0.003
5	0.107	0.026	0.023	0.020	0.023	0.002
6	0.174	0.033	0.034	0.036	0.034	0.001
7	-	-	-	-	-	-
8	0.176	0.038	0.042	0.044	0.041	0.002
9	0.114	0.028	0.026	0.026	0.027	0.001
10	0.179	0.041	0.032	0.029	0.034	0.005
11	-	-	-	-	-	-
12	0.218	0.049	0.052	0.049	0.050	0.001
13	0.119	0.030	0.002	0.002	0.012	0.012
14	0.189	0.004	0.032	0.027	0.021	0.011
15	0.106	0.044	0.050	0.043	0.046	0.003
16	0.228	0.056	0.035	0.046	0.046	0.007
17	0.158	0.029	0.035	0.028	0.031	0.003
18	0.165	0.043	0.030	0.033	0.035	0.005
19	0.163	0.039	0.043	0.037	0.040	0.002

Table S3. Desirability indexes (DI) for bioinspired membranes.

Experiment	[CT] mg/mL	[F127] mg/mL	[SSD] mg/mL	[PIP] mg/mL	DI
1	10	10	0.050	0.050	0.0000
**2**	**20**	**10**	**0.050**	0.050	**0.9088**
3	10	20	0.050	0.050	0.0000
4	20	20	0.050	0.050	0.0000
5	10	10	0.200	0.050	0.0000
6	20	10	0.200	0.050	0.7648
7	10	20	0.200	0.050	0.0000
8	20	20	0.200	0.050	0.0000
9	10	10	0.050	0.200	0.0000
10	20	10	0.050	0.200	0.7678
11	10	20	0.050	0.200	0.0000
12	20	20	0.050	0.200	0.0000
13	10	10	0.200	0.200	0.0000
14	20	10	0.200	0.200	0.0000
15	10	20	0.200	0.200	0.0000
16	20	20	0.200	0.200	0.0000
17	15	15	0.125	0.125	0.0000
18	15	15	0.125	0.125	0.0000
19	15	15	0.125	0.125	0.0000

Fourier transform infrared spectroscopy (FTIR)

FTIR spectra presented in Figure S1 were used to characterize the matrix formed by CT, F127, and the formulations containing PIP and SSD. Analysis of these spectra provides information on the intermolecular interactions that occurred due to the incorporation of the compounds.

In the spectrum of CT alone, the band around 1650 cm^{-1} corresponds to the C=O stretching of the amide I group, while the band near 1550 cm^{-1} is associated with the N-H deformation mode (amide II). 72 Furthermore, the band at 1050-1020 cm^{-1} can be attributed to the C-O-C stretching vibrations present in the chitosan structure.

For F127, the intense band around 1100 cm^{-1} stands out, corresponding to the C-O-C stretching vibrations, characteristic of the ethylene oxide repeating unit. A band can also be identified at 2880 cm^{-1}, associated with the C-H stretching of the methyl and methylene groups.

With the incorporation of PIP into CT/F127/PIP and SSD into QT/F127/SSD, no clear signals of these compounds were observed in the FTIR spectra. This absence is explained, firstly, by the low concentration of PIP and SSD in the formulations, which significantly reduces the intensity of their characteristic bands, making them less noticeable. Furthermore, spectral overlap with major components, such as CT and Pluronic F127, makes it difficult to detect specific bands for these drugs, since their functional groups (such as amides, sulfonamides, and carboxyls) vibrate in spectral regions already dominated by intense matrix signals.

Finally, the spectrum of the optimized complete membrane of CT/F127/PIP/SSD shows that the characteristic signals of chitosan and Pluronic F127 remain predominant. However, specific bands of PIP and SSD are not clearly identified, as previously reported. Therefore, these results demonstrate that CT and F127 are the major components in the polymer matrix, presenting well-defined bands in all formulations analyzed. Therefore, although the incorporation of PIP and SSD occurred, the lack of clear identification of their bands suggests that other complementary analytical techniques, such as thermal analysis (TGA and DSC), would be more suitable for analyzing the presence of these compounds and evaluating their interactions with the polymer matrix.

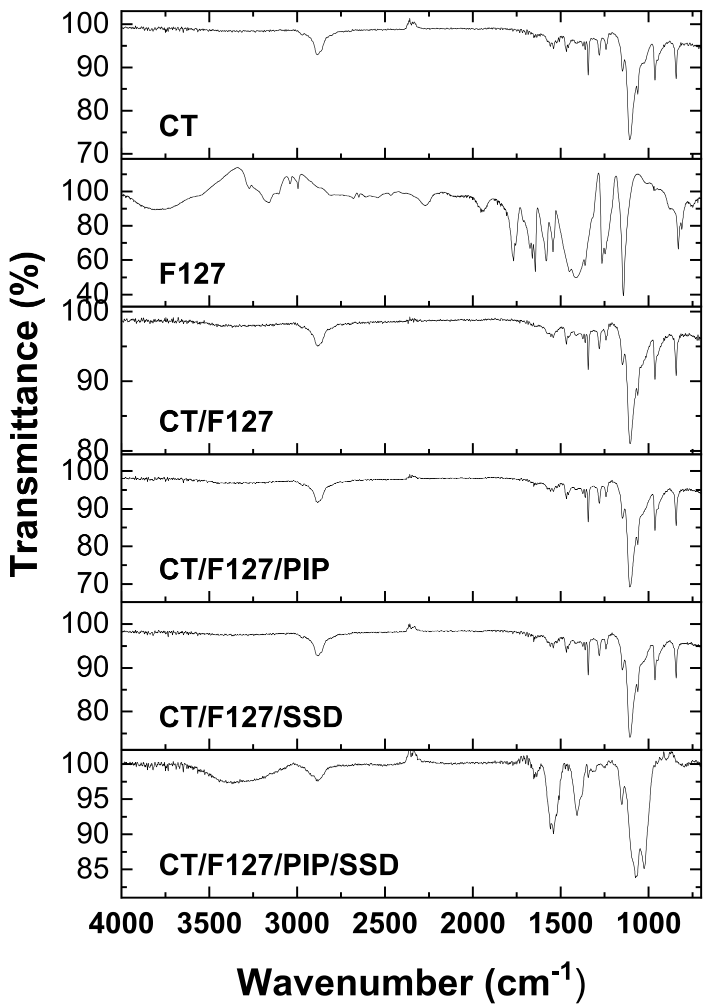

Figure S1. FTIR spectra obtained for the optimized membrane No. 2 (CT/F127/PIP/SSD) and its controls.

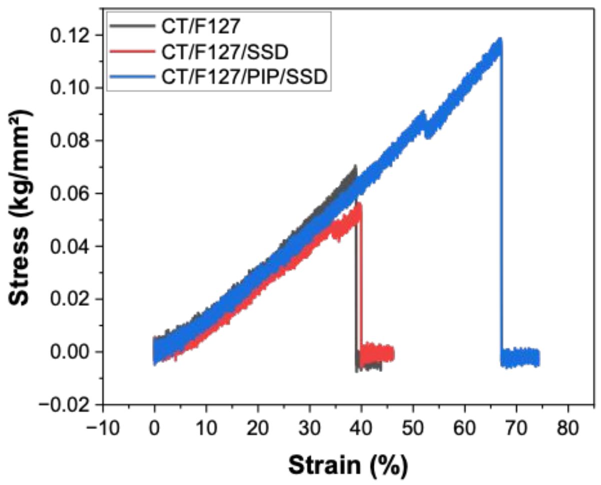

Figure S2. Stress–strain curves of chitosan/F127-based membranes with and without the incorporation of PIP and SSD after hydration.
